# CK-666 protects against ferroptosis and renal ischemia-reperfusion injury through a microfilament-independent mechanism

**DOI:** 10.1016/j.jbc.2024.107942

**Published:** 2024-10-29

**Authors:** Qian Hu, Yanan Zhao, Wan-yang Sun, Zexian Ou, Wentao Duan, Zeyu Qiu, Yuanlong Ge, Daolin Tang, Tianfeng Chen, Xiang Cheng, Rong-rong He, Shu Wu, Zhenyu Ju

**Affiliations:** 1 Key Laboratory of Regenerative Medicine of Ministry of Education, Department of Developmental & Regenerative Medicine, College of Life Science and Technology, Institute of Aging and Regenerative Medicine, Jinan University, Guangzhou, China; 2 Guangdong Engineering Research Center of Traditional Chinese Medicine & Disease Susceptibility, Guangzhou Key Laboratory of Traditional Chinese Medicine & Disease Susceptibility, Guangdong-Hong Kong-Macao Universities Joint Laboratory for the Internationalization of Traditional Chinese Medicine, Jinan University, Guangzhou, China; 3 School of Biomedical Sciences, Li Ka Shing Faculty of Medicine, the University of Hong Kong, China; 4 Department of Surgery, UT Southwestern Medical Center, Dallas, Texas, USA; 5 College of Chemistry and Materials Science, Jinan University, Guangzhou, China; 6 Department of Hematology, Children’s Hospital, Capital Institute of Pediatrics, Beijing, China

**Keywords:** ferroptosis, microfilament, lipid peroxidation, Arp2/3 complex, renal ischemia-reperfusion injury

## Abstract

Ferroptosis is a type of regulated cell death driven by iron-dependent accumulation of lipid peroxidation, exhibiting unique morphological changes. While actin microfilaments are crucial for various cellular processes, including morphogenesis, motility, endocytosis, and cell death, their role in ferroptosis remains unclear. Here, our study reveals that actin microfilaments undergo remodeling and disassembly during ferroptosis. Interestingly, inhibitors that target actin microfilament remodeling do not affect cell sensitivity to ferroptosis, with the exception of CK-666 and its structural analog CK-636. Mechanistically, CK-666 attenuates ferroptosis independently of its canonical function in inhibiting the Arp2/3 complex. Further investigation revealed that CK-666 modulates the ferroptotic transcriptome, prevents lipid degradation, and diminishes lipid peroxidation. In addition, CK-666 does not impact the labile iron pool within cells nor does the inhibition of FSP1 impacts its antiferroptosis activity. Notably, the results of DPPH assay and liposome leakage assay suggest that CK-666 mitigates ferroptosis by directly eliminating lipid peroxidation. Importantly, CK-666 significantly ameliorated renal ischemia-reperfusion injury and ferroptosis in renal tissue, underscoring its potential therapeutic impact.

Ferroptosis is a type of regulated cell death characterized by iron dependency and lipid peroxidation accumulation (1, 2), playing a critical role in various physiological and pathological conditions (3, 4). Chemicals that modulate ferroptosis hold significant therapeutic potential for numerous ferroptosis-related diseases, including ischemia-reperfusion injury (IRI), acute liver injury, and cancer (5). Ferroptotic cells exhibit unique morphological changes, such as aberrant mitochondrial structures and cellular rounding, yet maintain normal nuclear size without chromatin condensation or fragmentation (6).

Microfilaments, composed of actin filaments and associated proteins, are the thinnest and smallest components of the cytoskeleton in cells. Microfilaments are essential for cell morphology, motility, polarization, and endocytosis. These processes depend on the dynamic polymerization and depolymerization of actin, along with the varied architectures of actin networks (7). Formin, an actin-binding protein, orchestrates the nucleation and elongation of unbranched actin filaments (8). Concurrently, the Arp2/3 complex initiates branching at the side of the actin filaments, aided by nucleation-promoting factors such as N-WASP (9). The organization of actin filaments has been recognized to be crucial in different types of cell death including apoptosis and necroptosis (10, 11, 12). However, their involvement in ferroptosis remains less understood.

In our observations, cells undergoing ferroptosis display significant remodeling of actin microfilaments. Interestingly, drugs targeting actin dynamics, such as those affecting polymerization or branching, do not influence ferroptosis. Notably, CK-666, a recognized inhibitor of the Arp2/3 complex, effectively blocks ferroptosis but through mechanisms that are independent of its usual function in microfilament dynamics. Mechanistically, liquid chromatography-mass spectrometry analysis and various functional assays demonstrated that CK-666 mitigates ferroptosis through its hydrophobic antioxidant capacity. Additionally, CK-666 has shown to significantly mitigate ferroptosis-associated renal IRI *in vivo*, underscoring its potential as a therapeutic agent.

## Results

### Differential effects of actin cytoskeleton perturbators on ferroptosis

**Figure 1:**
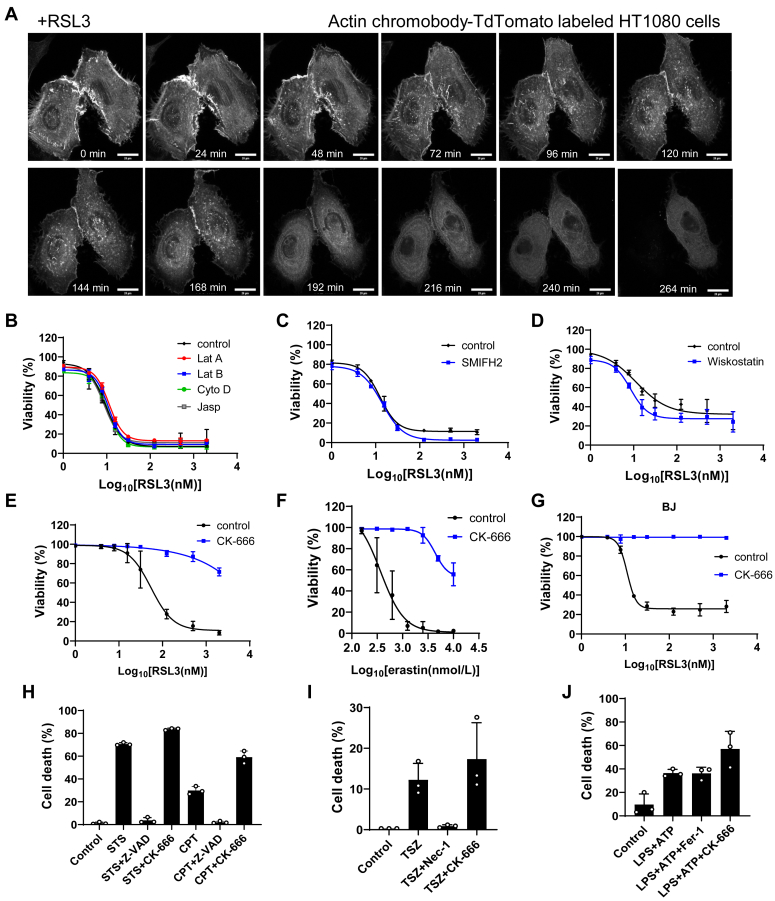
**Differential effects of actin cytoskeleton inhibitors on ferroptosis.***A*, live cell imaging of HT1080 cells labeled with actin chromobody-TdTomato. Frames at the indicated times after the addition of 2 μM RSL3 are shown (See also in Video S1). Scale bar, 20 μm. *B–D*, cell viability of HT1080 cells treated with or without Latrunculin A (Lat A, 1 μM), Latrunculin B (Lat B, 1 μM), cytochalasin D (Cyto D, 2 μM), jasplakinolide (Jasp, 0.04 μM), SMIFH2 (50 μM), or Wiskostatin (WS, 0.5 μM) in the presence of different concentrations of RSL3 for 24 h. *E*, cell viability of HT1080 cells treated with or without CK-666 (100 μM) in the presence of different concentrations of RSL3 for 24 h. *F*, cell viability of HT1080 cells treated with or without CK-666 (100 μM) in the presence of different concentrations of erastin for 24 h. *G*, cell viability of BJ fibroblasts treated with or without CK-666 (100 μM) in the presence of different concentrations of RSL3 for 24 h. *H*, cell death in HT1080 cells treated with CK-666 (100 μM) or Z-VAD (50 μM) for 24 h in conjunction with either Staurosporine (STS, 1 μM) or Camptothecin (CPT, 1 μM), both of which induce apoptosis. *I*, cell death in NIH3T3 cells treated with CK-666 (100 μM) or Nec-1s (50 μM) for 24 h in conjunction with TSZ (a mixture of 20 ng/ml TNF-α, 1 μM BV-6, and 50 μM Z-VAD), which induces necroptosis. *J*, cell death in bone marrow-derived macrophages treated with CK-666 (100 μM) or Fer-1 (2 μM) for 24 h in conjunction with a mixture of LPS (100 ng/ml) and ATP (5 mM), which induces pyroptosis. The values were presented as mean ± SD. n = 3 biologically independent replicates.

Actin filament remodeling has been shown to play significant roles in different types of cell death such as apoptosis and necroptosis (10, 11, 12). To examine the dynamics of actin during ferroptosis, we utilized a TdTomato-labeled actin chromobody in HT1080 cells. This chromobody specifically binds to actin, facilitating the real-time imaging of actin structures in living cells (13). Then, ferroptosis in cells was induced by RSL3, a commonly used ferroptosis inducer that inactivates glutathione peroxidase 4 (GPX4), a key suppressor of ferroptosis. Live cell imaging revealed progressive cell shrinkage, loss of adherence, and eventual rupture as cells underwent ferroptosis. Concurrently, dynamic changes in actin microfilaments were observed, culminating in the leakage of the chromobody postcell rupture (Fig. 1*A* and Video S1).

The basic dynamics of actin filaments are regulated by their polymerization and depolymerization. To further examine the role of microfilaments in ferroptosis, cells were exposed to various actin cytoskeleton perturbators, including Latrunculin A and B, which inhibit actin polymerization by binding to G-actin monomers (14), and Cytochalasin D, which impedes further polymerization at the barbed end of the actin filament (15). Conversely, Jasplakinolide stabilizes existing actin filaments and prevents their disassembly (16). None of these agents affected cell sensitivity to RSL3 (Fig. 1*B*). Additionally, treatment with SMIFH2, an inhibitor of formin which facilitates the elongation of unbranched actin filaments (17), did not modify sensitivity to ferroptosis (Fig. 1*C*). Similarly, inhibition N-WASP, which promotes actin filament branching as a nucleation-promoting factor with the assistance of the Arp2/3 complex, through Wiskostatin (18) did not impact cell ferroptosis (Fig. 1*D*). These findings indicate that although the actin cytoskeleton undergoes remodeling during ferroptosis, this process appears to be robust against disruptions to actin dynamics.

Interestingly, the small molecular inhibitor CK-666, which targets and stabilizes the inactive state of the Arp2/3 complex (19), significantly mitigated ferroptosis induced by both RSL3 (Figs. 1*E* and S1*A*) and erastin (Figs. 1*F* and S1*B*) in HT1080 cells. This inhibitory effect was also observed in BJ fibroblasts subjected to RSL3-induced ferroptosis (Fig. 1*G*). In contrast, when apoptosis (Fig. 1*H*), necroptosis (Fig. 1*I*), and pyroptosis (Fig. 1*J*) were induced in different cell lines, CK-666 did not confer protection, underscoring its specific action against ferroptosis.

### CK-666 attenuates ferroptosis independent of the Arp2/3 complex

**Figure 2:**
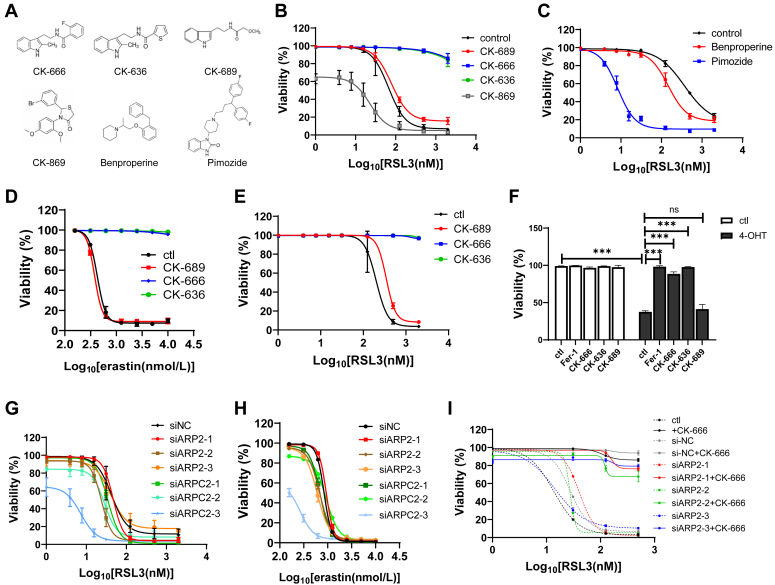
**CK-666 attenuates ferroptosis independent of the Arp2/3 complex.***A*, the molecular structures of CK-666, CK-636, CK-689, CK-869, Benproperine, and Pimozide. *B*, cell viability of HT1080 cells treated with or without CK-666 (100 μM), CK-636 (100 μM), CK-689 (100 μM), or CK-869 (100 μM) in the presence of different concentrations of RSL3 for 24 h. *C*, cell viability of HT1080 cells treated with or without Benproperine (10 μM) and Pimozide (10 μM) in the presence of different concentrations of RSL3 for 24 h. *D*, cell viability of HT1080 cells treated with or without CK-666 (100 μM), CK-636 (100 μM), or CK-689 (100 μM) in the presence of different concentrations of erastin for 24 h. *E*, cell viability of 293T cells treated with or without CK-666 (100 μM), CK-636 (100 μM), or CK-689 (100 μM) in the presence of different concentrations of RSL3 for 24 h. *F*, cell viability of Pfa1 cells treated with or without Fer-1 (2 μM), CK-666 (100 μM), CK-636 (100 μM), or CK-689 (100 μM) in the presence of 4-hydroxytamoxifen (4-OHT, 100 μM) for 72 h. *G*, cell viability of HT1080 cells transfected with siNC, siARP2, or siARPC2 in the presence of different concentrations of RSL3 for 24 h. *H*, cell viability of HT1080 cells transfected with or without siNC, siARP2, or siARPC2 in the presence of different concentrations of erastin for 24 h. *I*, cell viability of HT1080 cells treated with or without CK-666 (100 μM) and transfected with siNC or siARP2 in the presence of different concentrations of RSL3 for 24 h. The values were presented as mean ± SD. n = 3 biologically independent replicates.

We next explored whether CK-666 attenuates ferroptosis dependent of the Arp2/3 complex. The effect of other Arp2/3 complex inhibitors on ferroptosis were tested. CK-636, a structural analog of CK-666 with similar inhibitory effect on Arp2/3 complex, exhibited a similar ability to attenuate RSL3-induced ferroptosis (Fig. 2, *A* and *B*). In contrast, the inactivation control compound, CK-689, had no mitigating effect on ferroptosis (Fig. 2, *A* and *B*). Interestingly, CK-869, a methoxy group containing compound that inhibits the Arp2/3 complex through a distinct structural mechanism compared to CK-666 (20), slightly sensitized cells to RSL3 (Fig. 2, *A* and *B*). Moreover, neither Benproperine nor Pimozide (21, 22), representing alternative Arp2/3 complex inhibitors, showed any ability to inhibit ferroptosis (Fig. 2, *A* and *C*). To determine the consistency of the impact of CK-666/CK-636 on ferroptosis across various cell types and triggers, ferroptosis was induced in HT1080 cells using erastin (Fig. 2*D*), in 293T cells with RSL3 (Fig. 2*E*), and in Pfa1 cells through tamoxifen-induced GPX4 deletion (Fig. 2*F*). Remarkably, CK-666/CK-636 prevented ferroptosis under all tested conditions, whereas CK-689 did not exhibit any protective effects (Fig. 2, *D*–*F*). This suggests a selective efficacy of CK-666/CK-636 in rescuing cells from ferroptosis, in contrast to other Arp2/3 complex inhibitors.

It has been reported that ARPC4 knockout, which depletes the Arp2/3 complex, leads to NRF2 hyperactivation (23), which may block ferroptosis (24). In line with this, NRF2 inhibition *via* ML385 enhanced ferroptosis susceptibility (Fig. S2*A*). However, treatment with CK-666/CK-636 did not affect either total or phosphorylated NRF2 levels in HT1080 cells (Fig. S2*B*), and their protective effect against ferroptosis was not diminished by ML385 (Fig. S2*A*). Further examination involving knockdown of ARP2 and ARPC2, major components of the Arp2/3 complex, showed that neither deficiency protected cells from RSL3 or erastin-induced ferroptosis (Fig. 2, *G* and *H*). Notably, CK-666 continued to mitigate ferroptosis effects even when ARP2 or ARPC2 was suppressed (Fig. 2*I*). These results collectively suggest that the inhibitory effect of CK-666 on ferroptosis occurs independently of the Arp2/3 complex.

### CK-666 rescues ferroptosis signatures in transcriptome and lipidome

**Figure 3:**
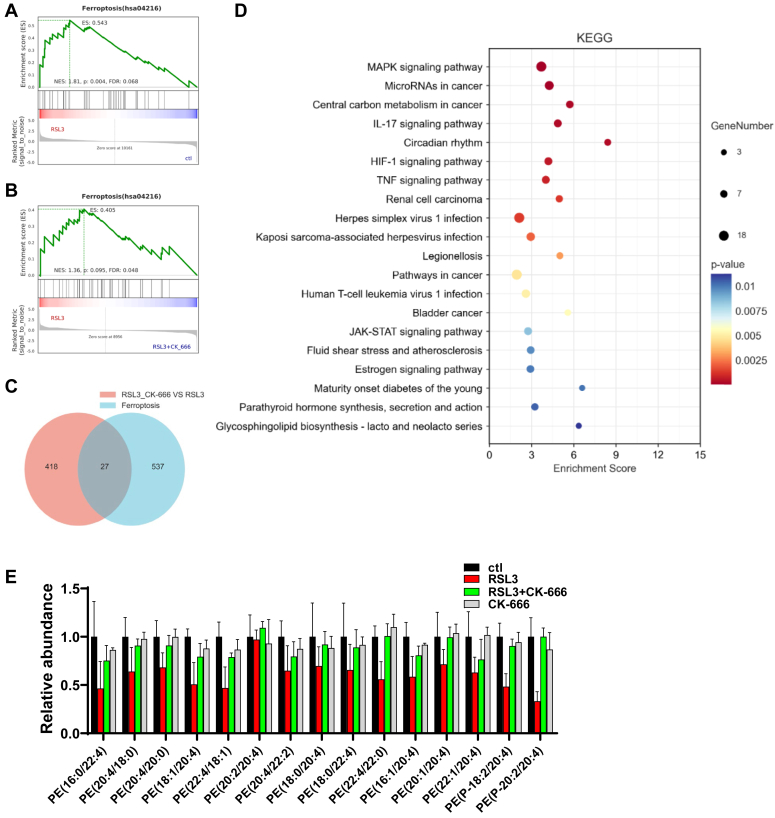
**CK-666 affects ferroptosis proteome and related HIF-1α signaling.***A*, gene set enrichment analysis (GSEA) for ferroptosis in HT1080 cells treated with RSL3 (2 μM) or not treated for 2 h. *B*, GSEA for ferroptosis in HT1080 cells treated with RSL3 (2 μM) and CK-666 (100 μM) for 3.5 h. *C*, Venn diagram of the co-expressed differentially expressed genes (DEGs) among RSL3 *versus* RSL3+CK_666 and ferroptosis genes. Ferroptosis-related genes were obtained from the FerrDb v2 database. The DESeq2 package was utilized to identify DEGs, with a significance threshold of *p* value < 0.05 and |log2FC| > 0.58. *D*, pathways enriched of DEGs from KEGG pathway enrichment analysis. *E*, relative abundance of phosphatidylethanolamines (PEs) containing arachidonic acid (20:4) and adrenic acid (22:4) in HT1080 cells treated with RSL3 (2 μM) and CK-666 (100 μM) or not treated for 3 h. KEGG, Kyoto Encyclopedia of Genes and Genomes.

To investigate the mechanism underlying CK-666's protective effect against ferroptosis, we performed RNA-seq analysis on HT1080 cells treated with CK-666. Gene Set Enrichment Analysis identified a significant upregulation of ferroptosis-related gene sets in cells treated with RSL3 compared to control (Fig. 3*A*). Interestingly, CK-666 treatment led to a general trend of downregulation in these ferroptosis-related genes (Fig. 3*B*). Specifically, there were 445 differentially expressed genes in the RSL3 plus CK-666 group compared to the RSL3 group alone, with 27 of these being ferroptosis-associated genes identified in the FerrDb V2 database (25) (Fig. 3*C*). Further, Kyoto Encyclopedia of Genes and Genomes enrichment analysis highlighted an activation of genes in the MAPK, HIF-1, and JAK-STAT signaling pathways following CK-666 treatment (Fig. 3*D*).

Lipidome analysis revealed a significant reduction in most phospholipids containing polyunsaturated fatty acids (PUFAs) upon RSL3 treatment, indicating their oxidation and subsequent degradation during ferroptosis (Fig. S3*A*). However, CK-666 treatment did not alter the levels of saturated fatty acids, monounsaturated fatty acids, or PUFAs compared to controls (Fig. S3*B*). Additionally, the levels of phosphatidylethanolamines containing arachidonic acid (20:4) and adrenic acid (22:4), key phospholipids in ferroptosis, remained unchanged with CK-666 treatment compared to control (Fig. 3*D*). This suggests that while CK-666 prevents the ferroptosis signature in the lipidome, it likely does not exert its protective effects through lipid remodeling. These findings indicate that CK-666 modulates ferroptosis-related gene expression and signaling pathways, albeit without directly influencing lipid remodeling associated with ferroptosis.

### CK-666 eliminates lipid peroxidation in cells through its hydrophobic antioxidant capacity

**Figure 4:**
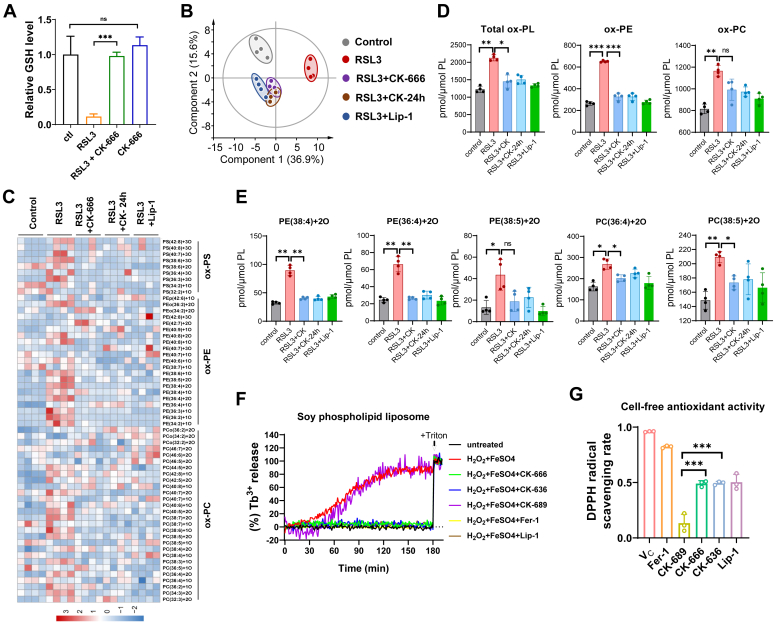
**CK-666 inhibits lipid peroxidation accumulation.***A*, relative GSH levels in HT1080 cells treated with CK-666 (100 μM) or RSL3 (2 μM) for 2 h. n = 3 biologically independent replicates. *B*, principal component analysis of oxygenated phospholipids in HT1080 cells treated with RSL3 (2 μM) for 3 h, RSL3 (2 μM) + CK-666 (100 μM) for 3 h, RSL3 (2 μM) + CK-666 (100 μM) for 24 h, and RSL3 (2 μM) + Lip-1 (1 μM) for 3 h. n = 4 biologically independent replicates. *C*, heatmap showing the relative changes of oxygenated PC, PE, and PS molecular species in the samples described in (*B*). *D*, levels of total oxygenated phospholipid (PL), PE, and PC in samples described in (*B*). *E*, levels of different types of PE or PC that contains arachidonic acid (20:4) or adrenic acid (22:4) in samples described in (*B*). *F*, Tb^3+^ release from soy phospholipid liposomes treated with CK-666 (100 μM), CK-636 (100 μM), CK-689 (100 μM), Fer-1 (2 μM), or Lip-1 (1 μM) in the presence of H_2_O_2_ (10 μM) and FeSO_4_ (50 μM). *G*, cell-free antioxidant activity of Vitamin C (Vc, 10 μM), Fer-1(10 μM), CK-689 (500 μM), CK-666 (500 μM), CK-636 (500 μM), and Lip-1 (5 μM) quantified by DPPH radical scavenging rates. n = 3 biologically independent replicates. The values were presented as mean ± SD. Statistical significance is indicated as follows: ns, not significant; ∗*p* < 0.05, ∗∗*p* < 0.01, ∗∗∗*p* < 0.001. GSH, glutathione; PC, phosphatidylcholine; PE, phosphatidylethanolamines.

Next, we explored the protective mechanism of CK-666 against ferroptosis. The results showed that CK-666 prevented glutathione (GSH) depletion induced by RSL3 (Fig. 4*A*). To investigate the impact of CK-666 on lipid peroxidation in cells, we utilized the fluorescent probe C_11_-BODIPY to determine lipid peroxidation levels. Surprisingly, CK-666/636 treatment rescued cells from RSL3-induced ferroptosis yet maintained high oxidative levels of C_11_-BODIPY, comparable to controls (Fig. S4*A*). This was corroborated by similar findings using Liperfluo, another lipid peroxidation probe (26) (Fig. S4*B*). Notably, in Pfa1 cells treated with 4-hydroxytamoxifen, the oxidative levels of C_11_-BODIPY remained elevated even with CK-666/636 intervention (Fig. S4*C*). These probes, while sometimes exhibiting antioxidant properties that can skew results (27, 28), consistently showed high levels of lipid oxidation, challenging their reliability under certain conditions. To address this, we conducted a titration of ferroptosis inhibitors, finding that 100 μM CK-666, a routinely used concentration (9), was as effective as 31 nM ferrostatin-1 (Fer-1) and 12.5 nM Lip-1 in mitigating ferroptosis in HT1080 cells, yet all treatments displayed similar levels of oxidative C_11_-BODIPY (Fig. S4*E*).

To accurately measure lipid peroxidation in cells, we conducted LC-MS-based redox phospholipidomics to examine oxygenated phospholipids (29). Principal component analysis indicated that treatment with CK-666 resulted in oxidized phospholipids with a composition similar to that of Lip-1 (Fig. 4*B*). Furthermore, RSL3 treatment induced a significant increase in oxygenated phospholipids in the cells, which was effectively mitigated by both CK-666 and Lip-1 (Fig. 4, *C* and *D*). Notably, CK-666 was comparable to Lip-1 in reversing the oxidation of phosphatidylethanolamines and phosphatidylcholines (PCs) containing PUFAs such as arachidonic acid (20:4) or adrenic acid (22:4) (Fig. 4*E*). Thus, CK-666 efficiently prevents the accumulation of lipid peroxidation in cells.

Ferroptosis is mediated by iron-dependent lipid peroxidation, and the level of labile iron pool significantly affect ferroptosis. Thus, we next measured the labile iron pool in cells treated with CK-666 using calcein, a dye whose fluorescence is quenched upon chelation by labile iron. The extent of quenching reflects the amounts of labile iron. Our results showed that CK-666 does not alter the iron levels in cells, indicating that its ferroptosis-suppressing activity is not dependent on iron modulation (Fig. S5*A*). Furthermore, we explored whether CK-666 inhibit ferroptosis relies on FSP1, a critical ferroptosis suppressor besides GPX4. Using iFSP1 (30) and viFSP1 (31), two inhibitors of FSP1, we found that these agents do not diminish CK-666's protective effect against cell ferroptosis, indicating that CK-666 acts independently of FSP1 (see Fig. S5*B*).

Next, we conducted a liposome leakage assay to investigate the effect of CK-666/636 on membrane oxidative rupture in a cell-free system (32). This assay involved enclosing Tb3+ within liposomes, composed of soy phospholipids containing PUFA, and triggering rupture through Fenton reaction-mediated lipid peroxidation. Fluorescence signals of chelates generated by dipicolinic acid and released Tb^3+^ were then detected. CK-666/636 protected the liposomes from oxidative rupture as effectively as Fer-1 and Lip-1 (Fig. 4*F*), suggesting their function independent of cellular proteins or metabolites.

Both CK-666 and CK-636 are characterized by their aromatic rings or conjugated systems allowing for electron delocalization. This structural feature may stabilize the molecules after their interaction with free radicals, effectively halting the propagation of chain reactions in lipid peroxidation (Fig. 2*A*). To assess the potential of CK-666 as a radical trapping agent, we utilized the DPPH assay. The results confirmed that both CK-666 and CK-636 scavenged radicals effectively, similar to known antioxidants Fer-1 and Lip-1, while CK-689 showed no such activity (Fig. 4*G*).

Overall, these results demonstrate that CK-666 mitigates lipid peroxidation in cells primarily through its hydrophobic antioxidant capacity, highlighting a potent mechanism by which CK-666 confers protection against ferroptosis.

### CK-666 alleviates renal IRI

**Figure 5:**
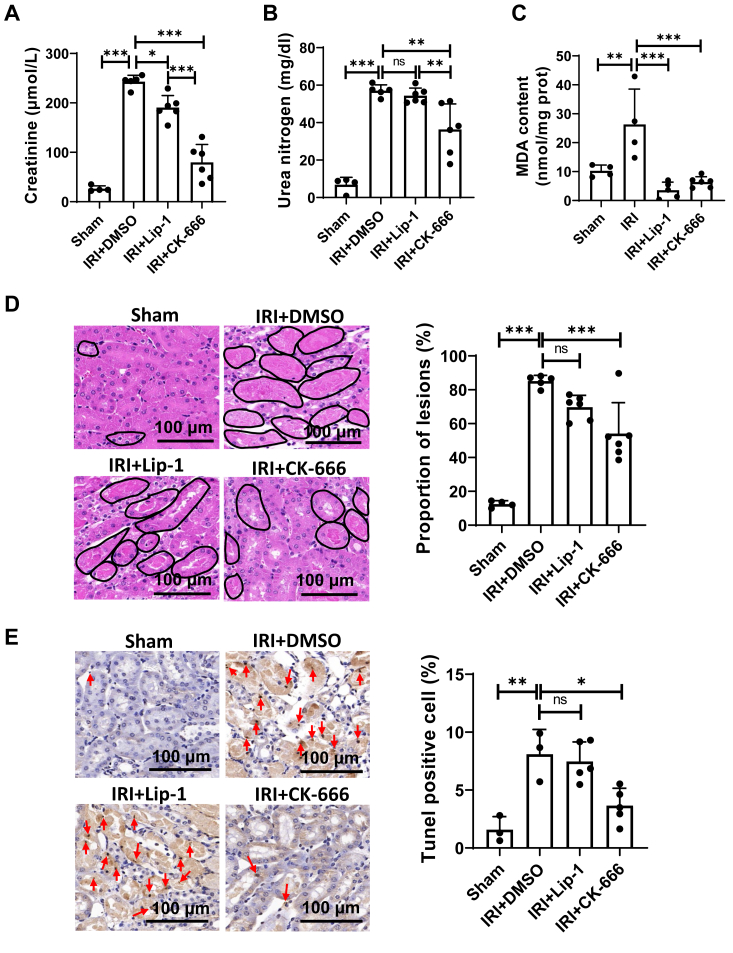
**CK666 alleviates renal ischemia-reperfusion injury.***A*, serum creatine levels in sham or renal ischemia-reperfusion injury (IRI) mice, intraperitoneally injected with Lip-1 (10 mg/kg) or CK-666 (30 mg/kg) were measured. n = 4 to 6 mice. *B*, serum urea nitrogen levels in sham or IRI mice treated as described in (*A*). n = 4 to 6 mice. *C*, MDA contents in the renal tissue of sham or IRI mice treated as described in (*A*). n = 4 to 6 mice. *D*, *left panel*: representative images of H&E staining of kidney sections in mice. *Right panel*: quantification of the proportion of lesions observed in H&E stained kidney sections. n = 4 to 6 mice. *E*, *left panel*: representative images of TUNEL staining of kidney sections in mice. *Red arrows* indicate TUNEL-positive cells. *Right panel*: quantification of TUNEL-positive cells in the kidney sections. n = 3 to 5 mice. The values were presented as mean ± SD. Statistical significance is indicated as follows: ns, no significance, ∗*p* < 0.05, ∗∗*p* < 0.01, ∗∗∗*p* < 0.001. MDA, malondialdehyde.

To further investigate the function of CK-666 *in vivo*, we induced ferroptosis in the kidneys of mice through renal ischemia-reperfusion. Postinjury, significant increases were observed in both serum creatinine and urea nitrogen levels (Fig. 5, *A* and *B*). Remarkably, CK-666 treatment effectively reduced these markers, suggesting an alleviation of kidney dysfunction (Fig. 5, *A* and *B*). Additionally, the levels of malondialdehyde, a biomarker of lipid peroxidation, were substantially elevated in renal tissues following ischemia-reperfusion. This increase was reversed by both Lip-1 and CK-666, confirming their protective effects against lipid peroxidation (Fig. 5*C*). Histological analyses using hematoxylin and eosin staining of renal tissues revealed significant tubular injuries postischemia, which were notably lessened by treatments with either Lip-1 or CK-666 (Fig. 5*D*). Furthermore, results from the TUNEL assay, which detects DNA fragmentation indicative of cell death, showed a marked increase in TUNEL-positive cells following ischemia-reperfusion. Interestingly, while Lip-1 treatment did not significantly reduce the number of dead cells, CK-666 treatment resulted in a substantial decrease in TUNEL-positive cells (Fig. 5*E*). These findings demonstrate the potent efficacy of CK-666 in mitigating renal IRI and ferroptosis, highlighting its therapeutic potential for renal protection.

## Discussion

Previous reports have linked various types of cell death to changes in cytoskeleton filaments. The early stages of apoptosis are associated with actin depolymerization, while microfilaments are redistributed yet largely preserved during autophagy-induced cell death (33). Further studies have demonstrated that the actin cytoskeleton participates in morphological alterations such as membrane blebbing and the formation of apoptotic bodies during apoptosis (34). Manipulation of actin filaments using agents like jasplakinolide or cytochalasin D induces apoptosis (35, 36, 37). During necroptosis, actin is involved in the trafficking of MLKL to the cell membrane (12). In our study, we observed the remodeling and disassembly of actin microfilaments during ferroptosis. However, disruptors of actin filaments did not interfere with ferroptosis as they do in apoptosis or necroptosis, suggesting a distinct regulatory mechanism.

Here, we elucidate the mechanism of action behind the ferroptosis-suppressing activity of CK-666. Initially, we investigated the role of the Arp2/3 complex and determined that CK-666 affects ferroptosis independently of its canonical function on this complex or NRF2. Subsequently, we performed comprehensive transcriptomic, lipidomic, and redox phospholipidomics analyses. These studies demonstrated that CK-666 effectively mitigates ferroptosis signatures, though the precise mechanism remains unclear. Furthermore, we explored the roles of labile iron and FSP1 in CK-666's function. Our findings confirmed that CK-666 does not alter the labile iron pool in cells nor does FSP1 inhibition impact its ability to suppress ferroptosis. Notably, results from the DPPH assay underscored CK-666's robust antioxidant activity. Most importantly, CK-666 prevented the leakage of oxidized liposomes, indicating its function independent of cellular proteins or metabolites. In summary, these findings suggest that the antiferroptotic effect of CK-666 is primarily attributed to its hydrophobic antioxidant capacity.

Both C_11_-BODIPY and Liperfluo are hydrophobic fluorescent dyes that are incorporated into lipid membranes and indirectly sense ROS, allowing the detection of lipid peroxidation. Oxidation of the polyunsaturated butadienyl segment of C_11_-BODIPY causes a shift in fluorescence emission with a high quantum yield (38). The oxidized form of Liperfluo emits green fluorescence in lipophilic sites and is nearly nonfluorescent in aqueous environment, thus providing specificity to lipid peroxides while minimizing photodamage and autofluorescence (26). However, these dyes also exhibit antioxidant properties that convert lipid hydroperoxides into lipid alcohols, potentially overestimating lipid peroxidation and underestimating the antioxidant effects of tested reagents (27, 28). Consistently, our findings show elevated levels of the oxidized forms of C_11_-BODIPY and Liperfluo despite CK-666 inhibiting cell ferroptosis. Similar outcomes were observed when concentrations of Fer-1 and Lip-1 were adjusted to inhibit ferroptosis at levels comparable to CK-666. LC-MS, despite its low throughput and technical challenges, directly analyzes specific oxidized lipids and is considered the most accurate method for measuring lipid peroxidation. Our LC-MS analysis revealed that lipid peroxidation is reduced in cells treated with CK-666. Therefore, our study suggests that when using fluorescent probes to assess lipid peroxidation during ferroptosis, the concentration of antioxidant agents should be carefully titrated.

The Arp2/3 complex is crucial for the branching of actin filaments involved in processes such as cell migration, endocytosis, and necessary morphological changes for various cellular functions. Inhibitors of the Arp2/3 complex, including benproperine phosphate and pimozide, have been used clinically for their antitussive and antipsychotic effects, respectively. CK-666, primarily functioning to inhibit the Arp2/3 complex, protects against methamphetamine-induced occludin internalization and increases transendothelial monocyte migration in mice (39). Additionally, CK-666 exhibits antifibrotic properties in the lung *in vivo* (40). Here, we found that CK-666 rescues cells from ferroptosis, dependent on its hydrophobic antioxidant capacity rather than its Arp2/3 complex inhibition effect. Functionally, CK-666 inhibits ferroptosis and protects against IRI *in vivo*. The potential of CK-666 as a therapeutic agent for ferroptosis-related diseases warrants further pharmacokinetic testing.

## Experimental procedures

### Mice

C57BL/6 WT mice were purchased from GemPharmatech Company. Two- to four-months age mice of both sexes were utilized, and all experimental protocols were approved by the Animal Care and Ethics Committee of Jinan University.

### Cell culture

HT1080 cells, 293T cells, BJ fibroblasts, pfa1 cells, NIH3T3 cells, and bone marrow-derived macrophages were cultured in Dulbecco’s modified Eagle’s medium (Gibco) supplemented with 10% FBS (Gibco) and 1% penicillin/streptomycin (Gibco) at 37 °C with atmosphere of 5% CO_2_. Bone marrow-derived macrophages were differentiated from into mouse bone marrow cells upon treatment with 20 ng/ml M-CSF (PerproTech) for 7 to 9 days.

### Chemicals

The compounds used to treat cells are as follow: RSL3, erastin, CK636, Ferrostatin-1, Liproxstatin-1, Necrostatin-1, and Z-VAD-FMK were purchased from Selleck; Wiskostatin, ML385, iFSP1, Benproperine phosphate, and Pimozide were purchased from Targetmol; and Deferoxamine and viFSP1 (MCE), CK-666 (Abcam), CK-869 (Macklin), CK-689 (Merck), Jasplakinolide (Invitrogen), Cytochalasin D (Aladdin), Latrunculin A (APE x BIO),Latrunculin B (Cayman), SMIFH2 (Sigma), and 4-Hydroxytamoxifen (Sigma).

### Measurement of lipid ROS with C11-BODIPY and Liperfluo

Cells were seeded on 6-well plate. After treated with compounds for indicated times, cells were digested by trypsin and washed by PBS. Then, cells were resuspended in 200 μl PBS containing 5 μM C_11_-BODIPY (Life Technology) or 1 μM Liperfluo (Dojindo). Incubate cells at 37 °C for 30 min. After washing with PBS, cells were stained with DAPI and detected by flow cytometry with BD Fortessa. The levels of lipid ROS were measured in the FITC channel.

### siRNA-mediated gene knockdown

siRNAs targeting ARPC2 (5′-CAGGGCAGUUAUCCAUUAU-3′, 5′-GGUCCUCUAUCAUAUUUCA-3′, 5′-GGCCUAUAUUCACACACGU-3′) and ARP2 (5′-GGCACCGGGUUUGUGAAGU-3′, 5′-TGGTGCTTCAAATCTCTCTCC-3′, 5′-CAGAAACTGGCCTTAGAAA-3′) were designed and synthesized by GenePharma (China). HT1080 cells were cultured into a 6-well plate in Dulbecco’s modified Eagle’s medium with 10% (v/v) FBS and 1% (v/v) penicillin/streptomycin and reach 70% confluent at transfection. siRNAs were mixed with Lipofectamine RNAiMAX (Life Technology) Transfection Reagent in Opti-MEM medium (Life Technology) according to the manufacturer’s instruction. Then the mixture was gently added to the medium. The culture medium was refreshed by siRNA-free medium after 12 h incubation. Cells were then transferred into a 96-well plate the next day for viability measurement.

### Cell viability measurement

Briefly, cells were cultured into a 96-well plate. After treatment with different compounds, Hoechst 33,342 and PI were added to stain total cell and dead cell, respectively. Then, the cell images were captured, and the viability was measured with ImageXpress Micro Confocal (Molecular Devices).

### DPPH assay

The radical trapping activity of chemicals was assessed using DPPH radical Scavenging Assay Kit (Solarbio, BC4755) following the manufacturer’s instruction. Briefly, compounds in extracting solution were added to DPPH and rotated for 30 min at room temperature. Absorbance at 412 nm was detected by a microplate reader (Synergy H1, BioTek).

### Labile iron pool measurement through calcein

Cells were treated with drugs for 24 h and then trypsined. After PBS washing, cells were stained with calcein-AM (0.25 μM) for 10 min at 37 °C. Calcein-AM passes the plasma membrane and reacts with cytosolic esterases. The fluorescence of calcein, the reaction products, is quenched following chelation of labile iron. After PBS washing, the cells were suspended in PBS. Labile iron pools were measured using the FITC channel by flow cytometry with Fortessa (BD).

### Western blot

Cell pellets and tissues were lysed in RIPA buffer plus the mixture of protease inhibitors and phosphatase inhibitors. Then, the protein concentrations were determined by BCA protein assay kit (Thermo). The proteins were run on SDS-PAGE gels and transferred onto PVDF membranes (Bio-Rad). Antibodies against GPX4 (Abcam, ab125066), ARP2 (CST, 3128T), ARPC2 (Abcam, ab133315), NRF2 (Abcam, ab62352), β-actin (ABclonal, AC026), and GAPDH (CST, 5174) were used. Signals were detected on an Amersham Imager 600 System (GE Health). The data were analyzed using ImageJ.

### GSH level measurement

GSH level measurement was performed using Total Glutathione Assay Kit (S0053, Beyotime). Cells were seeded into 12-well plates (1.5 × 10^5^ per well). After drug treatment, cells were collected by scraping and prepared for measurement of GSH according to the manufacturer’s instruction.

### Liposome leakage assay

Liposome leakage assay was performed as previously described (25). To prepare liposomes, 2 mg of soy phospholipid (Avanti) dissolved in 500 μl chloroform was transferred into a round bottomed flask. A thin lipid film was formed after chloroform was evaporated by flask spinning (150 rpm) and nitrogen purging. One milliliter buffer TL containing 1 M Hepes (pH 7.4), 5M NaCl, 2M sodium citrate, and 1.5 M TbCl_3_ was added into the flask. The lipid film was detached by vortex. Then, the lipid film was extruded 30 times by an Avanti Mini-Extruder (Avanti) with 100 nm polycarbonate membranes to generate morphologically uniform liposomes. To remove unencapsulated Tb3^+^ ions, liposomes were washed with buffer L containing 1M Hepes (pH 7.4) and 5M NaCl in a 100 KD cut-off ultrafiltration tube (Millipore) for at least 8 times. The collected liposomes were used for liposome leakage assays. Hydrogen peroxide (10 μM) and 50 μM FeSO_4_ were used to induce liposome leakage through Fenton reaction. Compounds to be tested were mixed with liposomes before the addition of hydrogen peroxide and FeSO_4_. To assess liposome leakage, the release of Tb3^+^ was detected by the addition of 50 μM of dipicolinic acid, and the fluorescent signals were measured through a microplate reader (λex = 270/λem = 620 nm).

### Living cell imaging

HT1080 cells were infected with Lentivirus HBLV-actin chromobody-TdTomato-puro (HANBIO). After puromycin treatment for 2 days, HT1080 cells expressed actin chromobody-TdTomato were obtained. Live cell imaging was acquired with Zeiss LSM 880 equipped with a definite focus system. Movies were analyzed with ImageJ.

### Lipidomic analysis

Homogenize cell sample with 1 ml mixture of methanol, MTBE, and internal standard. After centrifugation, 500 μl supernatant was separated and then concentrated. Then dissolve samples with 100 μl mobile phase B. To perform UPLC, the samples were extracted through ACQUITY UPLC HSS T3 C18 column (Waters). Next, LIT and triple quadrupole (QQQ) scans were acquired on a triple quadrupole-linear ion trap mass spectrometer (QTRAP), QTRAP LC-MS/MS System, equipped with an ESI Turbo Ion-Spray interface, was operated in positive and negative ion mode and controlled by Analyst 1.6.3 software (Sciex). Instrument tuning and mass calibration were performed with 10 and 100 μmol/L polypropylene glycol solutions in QQQ and LIT modes, respectively. A specific set of MRM transitions was monitored for each period according to the metabolites eluted within this period.

### Analysis of oxidized phospholipids by LC-MS

Lipids were extracted using Folch method. A comprehensive analysis of oxidized phospholipidomics was conducted, followed previously established method (39). Briefly, samples were subjected to LC-MS analysis utilizing a Dionex Ultimate 3000 LC system coupled with a Q-Exactive mass spectrometer (Thermo Fisher). A Luna Silica (2) column (3 μm, 150 × 2.0 mm) (Phenomenex) was used to separate the samples at a flow rate of 0.2 ml/min at 35 °C. The gradient elution schedule was set according to the following parameters: 0 min, 10% B; 23 min, 32%; 32 min, 65%; 35 min, 100%; and 70 min, 100%. Ionization was performed in negative ion mode with a capillary spray voltage of −2.8 kV, while the capillary temperature was set at 320 °C. The S-lens Rf level was set to 60. Data were acquired at a resolution of 70,000 for the full MS scan and 17,500 for the MS/MS scan in data-dependent mode, with the scan range set at m/z 400-1800 and a maximum injection time of 200 ms using 1 microscan. For MS/MS analysis, a maximum injection time of 500 ms was utilized, with collision energy set to 24 eV and an isolation window of 1.0 Da.

Raw LC-MS data preprocess was conducted using MZmine 2.5.3^40^, following a custom analysis workflow and database. Peaks with a signal-to-noise ratio above 3 were searched and identified against an in-house oxidized PL database. To identify lipid species, features were matched to m/z values with a tolerance of 5 ppm, further refined by retention time, and verified *via* MS/MS analysis using fragments for identification (https://www.lipidmaps.org/). Quantitative analysis relied on calibration curves created using known quantities of reference standards, including PA(18:1/18:1), PC(18:1/18:1), PE(18:1/18:1), PG(18:1/18:1), PI(18:1/18:1), PS(18:1/18:1), CL(16:0/18:2/18:2/20:4), and their corresponding internal standards PA(16:0-d31/18:1), PC(16:0-d31/18:1), PE(16:0-d31/18:1), PG(16:0-d31/18:1), PI(16:0-d31/18:1), PS(16:0-d31/18:1), and CL(14:0/14:0/14:0/14:0).

### Renal ischemia-reperfusion

Mice were intraperitoneally injected with Lip-1 (10 mg/kg) or CK-666 (30 mg/kg) 24 h and 2 h before surgery. The mice were anesthetized, and bilateral renal pedicles were clamped for 35 min to induce renal ischemia-reperfusion injury, during which the body temperature of mice was maintained on a heat plate at 38 °C. The mice in the Sham group underwent the same procedures as those in the renal I/R group except for drug administration and clamping. The mice were sacrificed 24 h after surgery. Serum and tissue samples were collected for subsequent testing.

### Hematoxylin and eosin staining

The kidney tissues were fixed with 4% paraformaldehyde 24 h to preserve cellular structures. Following fixation, the tissues underwent a gradient alcohol dehydration process using an automated dehydrator. Subsequently, they were embedded in paraffin wax blocks. The embedded tissues were sectioned at a thickness of 4 μm. Paraffin sections were deparaffinized and stained with hematoxylin and eosin for nuclei and cytoplasm, respectively. Then, the slices were dehydrated and sealed, and the image information was collected after drying. Sections were then scanned with panoramic scanning (3D HISTECH) to obtain high-definition images. Three fields were randomly selected for each sample to calculate the proportion of injured renal tubules.

### TUNEL staining

The kidney tissues were fixed with 4% paraformaldehyde 24 h to preserve cellular structures. Following fixation, the tissues underwent a gradient alcohol dehydration process using an automated dehydrator. Subsequently, they were embedded in paraffin wax blocks. The embedded tissues were sectioned at a thickness of 4 μm. These paraffin sections were stained using a DAB (SA-HRP) TUNEL Cell Apoptosis Detection Kit (Servicebio) according to the manufacturer's instructions. Following staining, the sections were dehydrated, sealed, and allowed to dry. High-definition images of the sections were captured using panoramic scanning (3D HISTECH). Three fields of view of consistent size were randomly selected for each sample. The number of dead cells within these fields was meticulously counted.

### Statistics

FlowJo, version 10, was used to analyze the flow cytometric data. GraphPad Prism 8 was utilized for statistical analysis. Image J was used to analysis images and movies. The results are shown as mean ± SD. In order to compare the groups based on a single factor, we employed the one-way analysis of variance method. For the comparison between groups affected by two factors, we utilized the two-way analysis of variance method. To account for the issue of multiple comparisons, we conducted a *post hoc* test using either Tukey’s multiple comparison test or Bonferroni correction to determine significant differences among the groups (ns, not significant, ∗*p* < 0.05, ∗∗*p* < 0.01, ∗∗∗*p* < 0.001).

## Data availability

Data that support the findings of this study are available within the article and in supporting information.

## Supporting information

This article contains supporting information.

## Ethics statement

No human participants, human data, or human tissues are involved in this study.

## Conflict of interest

The authors declare no conflict of interest with the contents of this article.
