# Supplementary material for: CK-666 protects against ferroptosis and renal ischemia-reperfusion injury through a microfilament-independent mechanism
Source: J Biol Chem. 2024 Oct 29;300(12):107942. doi: 10.1016/j.jbc.2024.107942 (PMC11625328; doi:10.1016/j.jbc.2024.107942)
Supplement: Supplementary Figure legend and Video legend [file mmc1.docx]

**Supplementary video legends**

**Supplementary video. The dynamic of actin filaments during cell ferroptosis. Related to Figure 1a.** Live cell imaging of actin chromobody-TdTomato labeled HT1080 cells that were treated with 2 μM RSL3. Images were processed with maximum intensity projection. Scar bar, 20 μm.

**Supplementary figure legends**

**Supplementary figure 1. CK-666 inhibits ferroptosis induced by RSL3 or erastin.**

**a.** Representative images of HT1080 cells stained with Hoechst and PI after treatment with different concentrations of RSL3 for 24 h. Scar bar, 100 μm.

**b.** Representative images of HT1080 cells stained with Hoechst and PI after treatment with different concentrations of erastin for 24 h. Scar bar, 100 μm.

**Supplementary figure 2. CK-666 rescues ferroptosis independent of NRF2 and Arp2/3 complex.**

**a.** Cell viability of HT1080 cells treated with CK-666 (100 μM) or ML385 (5 μM) in the presence of different concentrations of RSL3 for 24 h.

**b.** Levels of phosphorylated NRF2 (p-NRF2) and total NRF2 proteins in HT1080 cells treated with CK-689 (100 μM), CK-666 (100 μM), or CK-636 (100 μM) for 24 h.

**c.** ARP2 protein levels determined by western blotting in HT1080 cells transfected with siNC or siARP2.

**d.** ARPC2 protein levels determined by western blotting in HT1080 cells transfected with siNC or siARPC2.

The values were presented as mean ± SD. n = 3 biologically independent replicates. Statistical significance is indicated as follows: ns, not significant; * P<0.05, **P<0.01, *** p < 0.001

**Supplementary figure 3. CK-666 affects ferroptosis lipidome.**

**a.** Heatmap representing lipidome analysis showing the relative levels of different types of lipids in HT1080 cells treated with RSL3 (2 μM) and CK-666 (100 μM) for 3 h.

**b.** Relative abundance of saturated fatty acids (SFA), monounsaturated fatty acids (MUFA) and polyunsaturated fatty acids (PUFA) in lipidome analysis.

Valuesare presented as mean ± SD. n = 3 biologically independent replicates.

**Supplementary figure 4. CK-666 rescues ferroptosis remaining oxidative fluorescent probes.**

**a.** Left panel: The representative flow cytometry results of C_11_-BODIPY staining of HT1080 cells treated with RSL3 (2 μM), CK-666 (100 μM), CK-636 (100 μM), DFO (100 μM), Lip-1 (1 μM), or Fer-1 (2 μM) for 2 h. Right panel: The statistics data of the proportion of oxidized C_11_-BODIPY (ox-C_11_) positive cells.

**b.** Relative mean fluorescence intensity (MFI) of oxidized Liperfluo (ox-Liperfluo) positive HT1080 cells treated with RSL3 (2μM), CK-666 (100 μM), CK-636 (100 μM), DFO (100 μM), Lip-1 (1 μM), or Fer-1 (2 μM) for 2 h.

**c.** Relative mean fluorescence intensity (MFI) of oxidized C_11_-BODIPY (ox-C_11_) in Pfa1 cells treated with 4-OHT (50 μM), CK-666 (100 μM), CK-636 (100 μM), DFO (100 μM), or Fer-1 (2 μM) for 48h.

**d.** Cell viability of HT1080 cells treated with different concentrations of Fer-1, Lip-1 or CK-666 in the presence of the indicated concentrations of RSL3.

**e.** Proportion of ox-C_11_ positive HT1080 cells treated with indicated concentrations of CK-666, Fer-1 or Lip-1 in the presence of 125 nM RSL3 for 4 h.

Values are presented as mean ± SD. n =3 biologically independent replicates. Statistical significance is indicated as follows: ns, not significant; * P<0.05, **P<0.01, p < 0.001

**Supplementary figure 5. CK-666 rescues ferroptosis independent on iron level and FSP1.**

**a.** MFI of calcein in HT1080 cells treated with FAC (100 μM), DFO (100 μM) or CK-666 (100 μM or 200 μM) for 24 h.

**b.** Cell viability of HT1080 cells treated with RSL3 (125 nM), iFSP (5 μM), viFSP1 (5 μM) or CK-666 (100 μM) for 24 h.

Values are presented as mean ± SD. n =3 biologically independent replicates. Statistical significance is indicated as follows: ns, not significant.
