# Supplementary figures and images for: CK-666 protects against ferroptosis and renal ischemia-reperfusion injury through a microfilament-independent mechanism
Source: J Biol Chem. 2024 Oct 29;300(12):107942. doi: 10.1016/j.jbc.2024.107942 (PMC11625328; doi:10.1016/j.jbc.2024.107942)

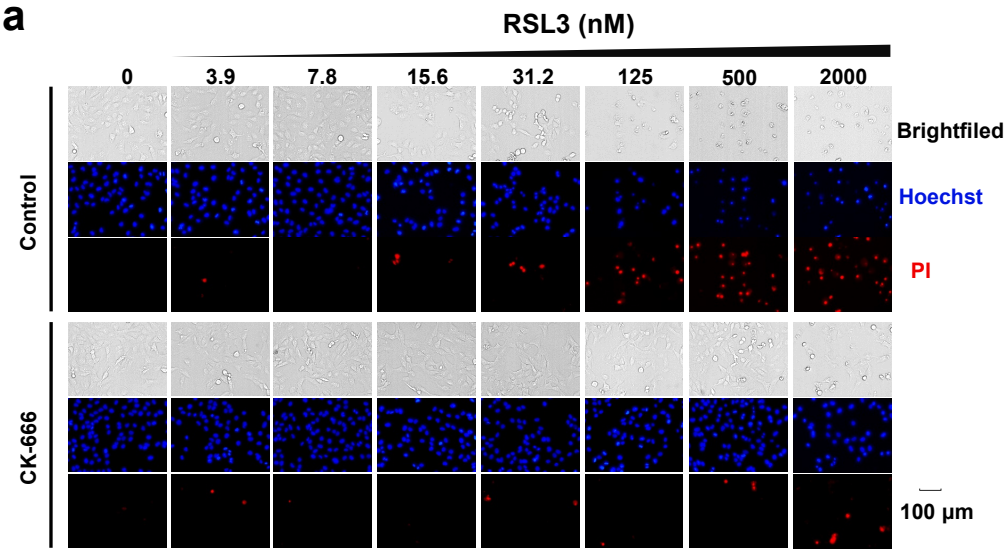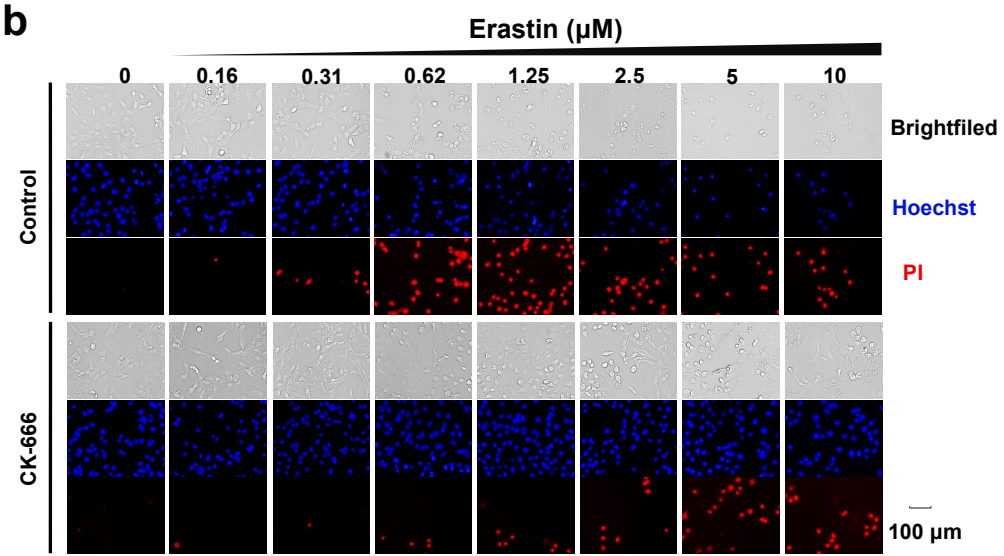

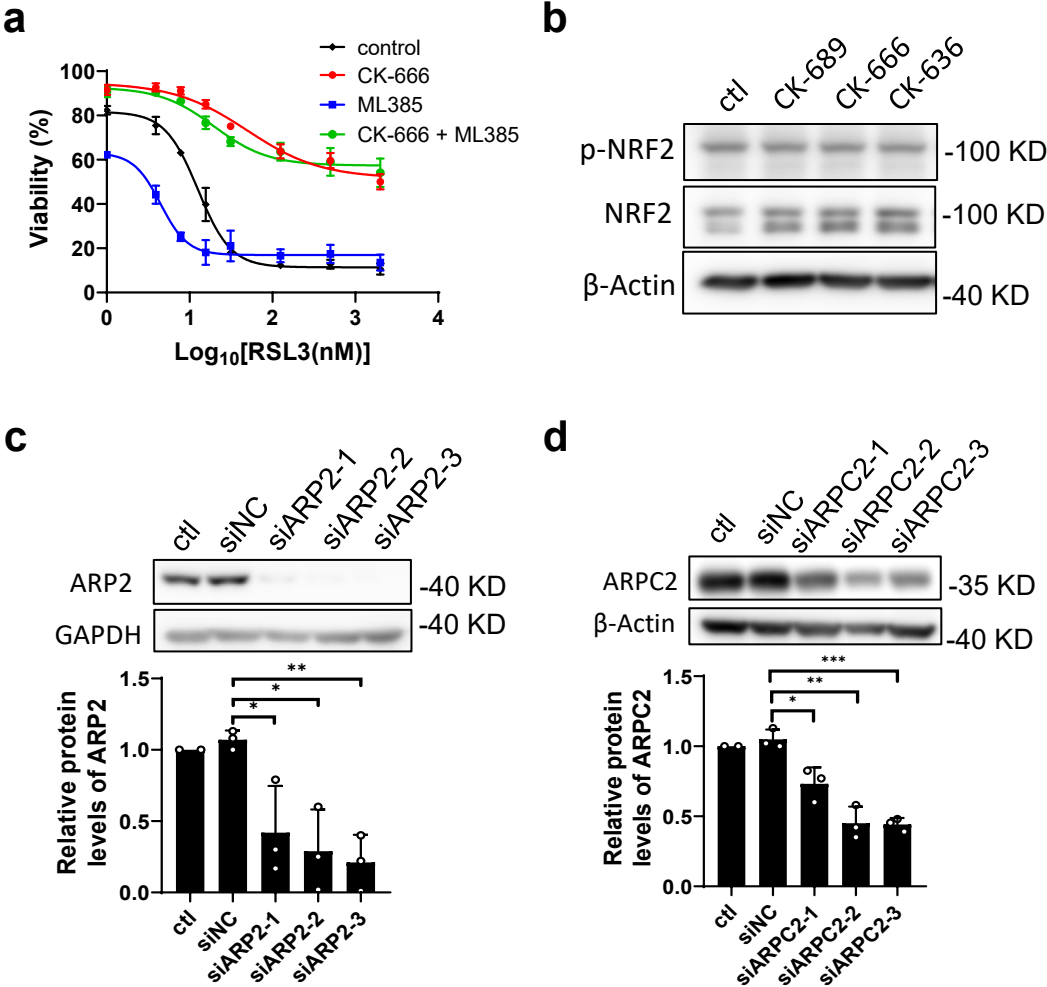

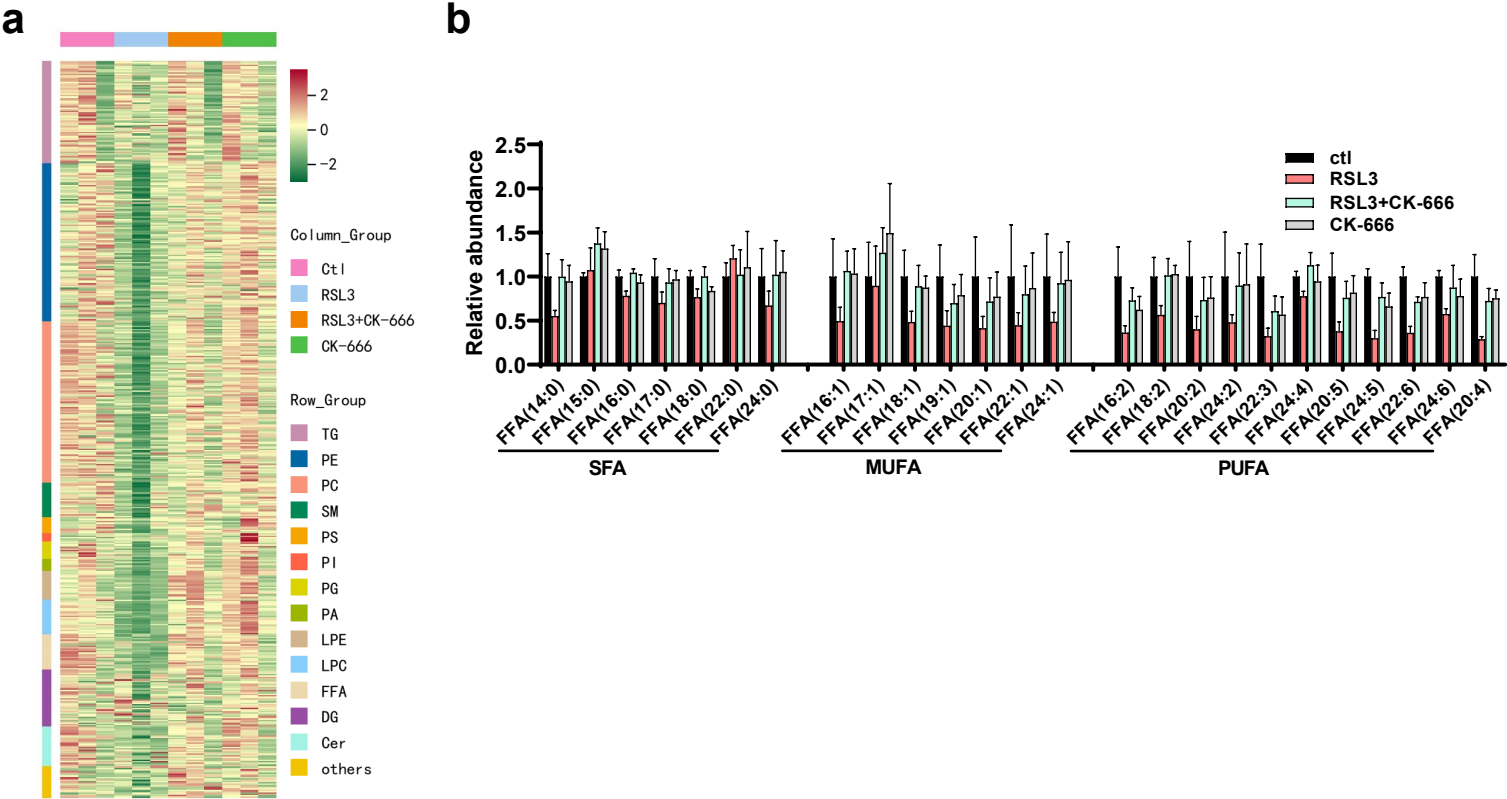

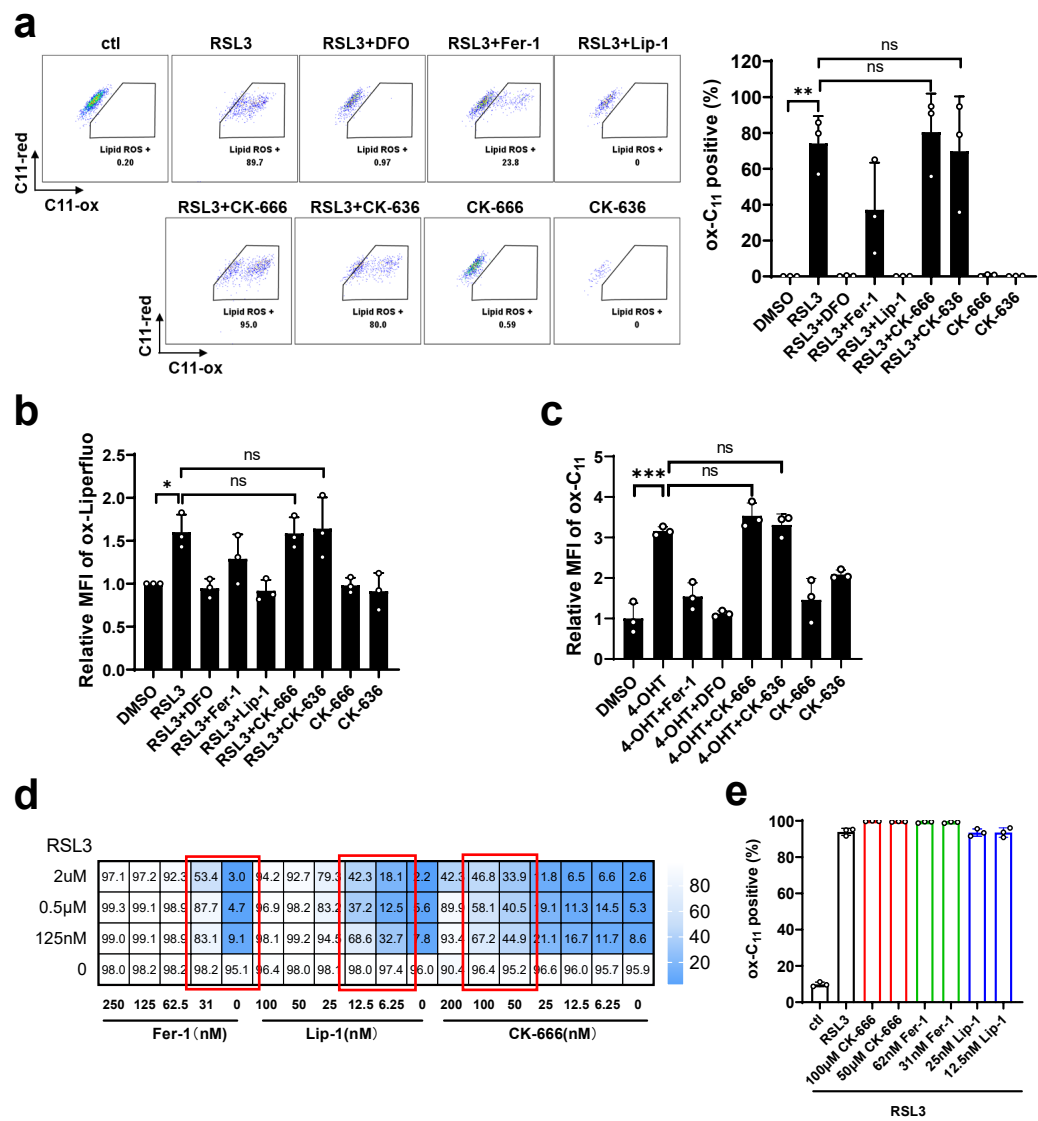

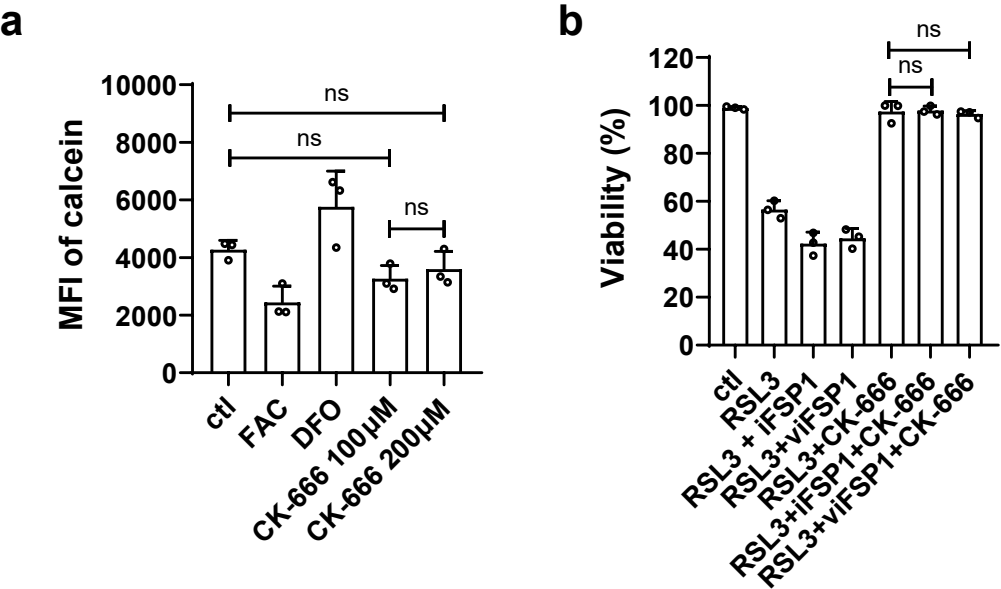

Supplement: Supplementary Figures [file mmc2.pdf]
